# *“Why has this new vaccine come and for what reasons?”* key antecedents and questions for acceptance of a future maternal GBS vaccine: Perspectives of pregnant women, lactating women, and community members in Kenya

**DOI:** 10.1080/21645515.2024.2314826

**Published:** 2024-02-12

**Authors:** Rupali J. Limaye, Prachi Singh, Berhaun Fesshaye, Clarice Lee, Jessica Schue, Ruth A. Karron

**Affiliations:** a Department of International Health, Department of Epidemiology, Department of Health, Behavior & Society, International Vaccine Access Center, Johns Hopkins Bloomberg School of Public Health, Baltimore, MD, USA; b Department of International Health, International Vaccine Access Center, Johns Hopkins Bloomberg School of Public Health, Baltimore, MD, USA; c Department of International Health, Johns Hopkins Bloomberg School of Public Health, Baltimore, MD, USA

**Keywords:** Pregnant women, lactating women, group B streptococcus, maternal immunization, Kenya

## Abstract

Group B streptococcus (GBS) is a leading global cause of neonatal sepsis and meningitis, stillbirth, and puerperal sepsis. While intrapartum antibiotic prophylaxis (IAP) is a currently available GBS disease prevention strategy, IAP is programmatically complex to implement, precluding use in low- and middle-income countries. In Kenya, 2% of stillbirths are attributable to GBS infection. Two maternal GBS vaccines are in late-stage clinical development. However, licensure of a maternal GBS vaccine does not translate into reduction of disease. We conducted 28 in-depth interviews with pregnant people, lactating people, and community members across two counties in Kenya to better understand the attitudes and informational needs of primary vaccine beneficiaries. We identified two emerging themes from the data. The first focused on antecedents to maternal GBS vaccine acceptability. The most common antecedents focused on the vaccine’s ability to protect the baby and/or the mother, followed by community sensitization before the vaccine was available. The second key theme focused on questions that would need to be addressed before someone could accept a maternal GBS vaccine. Three key categories of questions were identified, including vaccine safety compared to vaccine benefits, who gets the vaccine, and how the vaccine works. Realizing the potential benefits of a future GBS maternal vaccine will require a multifactorial approach, including ensuring that communities are aware of GBS-related harms as well as the safety and effectiveness of a maternal GBS vaccine. Our study contributes to informing this multifactorial approach by elucidating the attitudes and concerns of key populations.

## Introduction

Group B streptococcus (GBS) is a bacterium that colonizes the vaginal and gastrointestinal tracts of pregnant people.^1^ GBS colonization is usually asymptomatic among pregnant people, but can pass to the fetus or newborn in utero or during vaginal delivery.^1^ GBS is a leading global cause of neonatal sepsis and meningitis, stillbirth, and puerperal sepsis due to infants’ increased susceptibility to GBS infection and lower levels of immunity.^1,2^ Infants may experience GBS in two forms: early-onset disease, which occurs between birth and 6 days of life, or late onset, occurring between 7 and 89 days.^3^ Recent estimates are that there are approximately 230,000 early onset cases and 160,000 late onset cases occurring each year, resulting in 90,000 deaths and 37,000 survivors with neurodevelopmental impairment.^3^ In Kenya, the location of our study, 2% of stillbirths have been attributed to GBS infection.^4^

Two screening tools are used for identifying at-risk newborns for early onset GBS, microbiological and risk-factor screening; each has biological and implementation limitations.^5^ Microbiological screening is a collection of rectal and swabs followed by a culture or polymerase chain reaction (PCR). While microbiological screening is effective in identifying GBS disease among infants, low-resource settings lacking wide availability of diagnostic methods, are unable to implement it.^5^ Risk factor-based screening involves reviewing known risk factors in newborns, and while this approach is less costly than other screening approaches, studies have reported varying effectiveness.^5^ Despite the presence of these screening techniques, in Kenya, pregnant individuals are not routinely screened for GBS colonization.^6^ Intrapartum antibiotic prophylaxis (IAP) is the only currently available GBS disease prevention strategy and is used in at least 60 countries.^7^ The recommendation for IAP is administration of intravenous antibiotics for at least four hours before delivery to prevent vertical transmission of GBS.^8^ Although IAP has been effective in reducing early-onset disease in high income countries, it is programmatically complex to implement, precluding use in LMICs; moreover, IAP prevents early-onset GBS disease but is ineffective against late-onset disease. IAP may also not be feasible in some settings if a substantial number of births occur outside of a health center.^1^ Kenya currently has no policy for maternal GBS screening and IAP administration.^4^ Treatment for suspected and confirmed cases of GBS is antibiotic therapy, with penicillin being the first-line drug.^1^ However, antibiotic therapy, including IAP, raises the concern for antimicrobial resistance.^1^ For these reasons, there is need for new approaches to reduce harms related to GBS. The development of GBS vaccines has been identified as a priority by the World Health Organization.^9–12^

Related to maternal immunization broadly in Kenya, the only vaccine recommended during pregnancy is tetanus, with coverage still being suboptimal in some areas.^13^ According to the last Demographic and Health Survey, 75% of women with a live birth in the two years before the survey received sufficient tetanus toxoid injections to protect their baby against neonatal tetanus.^14^ Maternal vaccines are primarily administered at health facilities, including public and private facilities, as well as through faith-based organizations outreach and campaign outreach. All pregnant individuals in Kenya are eligible for a free maternal health care, known as Linda Mama, which includes antenatal care and vaccination.^15^ For information about maternal vaccines, pregnant individuals in Kenya tend to trust healthcare providers for education and recommendations.^13,16^

Currently, two maternal GBS vaccines are in late-stage clinical development^17^ and offer the potential to reduce global maternal and neonatal morbidity and mortality.^3^ Unfortunately, licensure of a maternal GBS vaccine does not guarantee reduction of disease, as acceptance and uptake are critical to realize the benefits of the vaccine; previous research has pointed to the importance of increasing acceptance to improve maternal vaccine uptake.^18^ Understanding the attitudes and informational needs of primary vaccine beneficiaries prior to introduction can inform demand generation strategies and potentially lead to increased acceptance and uptake. To best understand beneficiary attitudes, we conducted original socio-behavioral research, which was exploratory in nature. In this study, we explore key antecedents (attitudes and beliefs that inform behavior) and questions pregnant people, lactating people, and community members have about a maternal GBS vaccine in two counties in Kenya.

## Methods

This original research study was cross-sectional in nature. The study objective was to identify key themes that emerged related to perceptions of a potential maternal GBS vaccine, using theoretical constructs related to health behavior specifically related to vaccine decision-making. Given that vaccine decision-making does not occur in a vacuum, we sought to better understand attitudes and information needs about not only potential beneficiaries, but also peers that lived in their community. This qualitative study conducted in-depth interviews with pregnant people, lactating people, and community members, including community health volunteers and family members of pregnant and lactating people. We included both pregnant and lactating people because while a maternal vaccine would be given in pregnancy, given that the disease primarily affects infants, understanding attitudes of pregnant as well as lactating people will need to be taken into consideration for successful vaccine acceptance. Participants were recruited from two counties in Kenya, Nakuru (rural) and Mombasa (urban), and the sample was evenly split between the two counties.

Data were collected from August to September 2022. Semi-structured interview guides were pre-tested with pregnant people in Kenya and included questions on knowledge and awareness of GBS disease, as well as attitudes toward a new maternal GBS vaccine. Data collectors participated in a three-day ethics and protocol training. Participants were recruited from 18 health facilities with antenatal clinics, and facilities at each care level were included. Recruitment varied slightly based on patient volume at the facility. Generally, participants were approached consecutively upon arrival at waiting rooms in health facilities, with facility staff referring potential participants who may have been missed upon arrival. Informed oral consent was obtained from all participants, and all participants were 18 or older.

Interviews were conducted in Swahili or English and audio recorded. Recordings were then transcribed and translated to English by a team fluent in both languages. A five-member team applied a grounded theory approach to data analysis using Atlas.ti software. Through two rounds of open coding, a code list was generated, refined, and finalized. The team coded approximately 25% of the transcripts, convened to discuss emerging themes, then coded the remaining transcripts. This study received ethical approval from (Kenya Medical Research Institute) and (Johns Hopkins Bloomberg School of Public Health Institutional Review Board).

## Results

**Figure 1.**
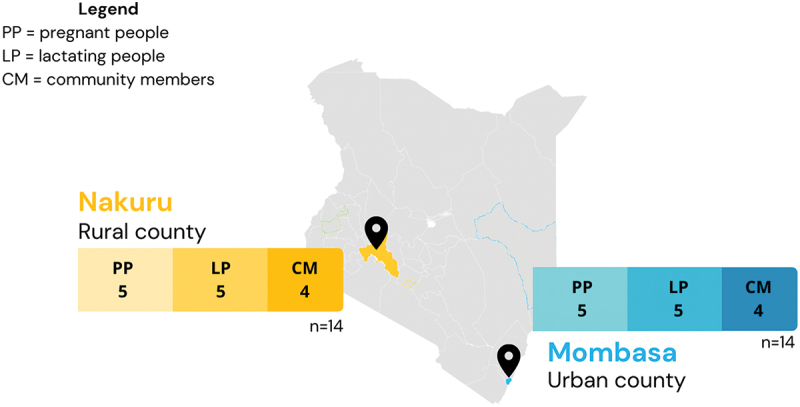
Map of sampled populations and locations across Kenya (*n* = 28 interviews; 10 pregnant people, 10 lactating people, 8 community members.

A total of 28 people were interviewed, with ten pregnant people, ten lactating people, and eight community members (see Figure 1). All pregnant and lactating participants identified as female, with two community members identifying as female and six identifying as male.

**Figure 2.**
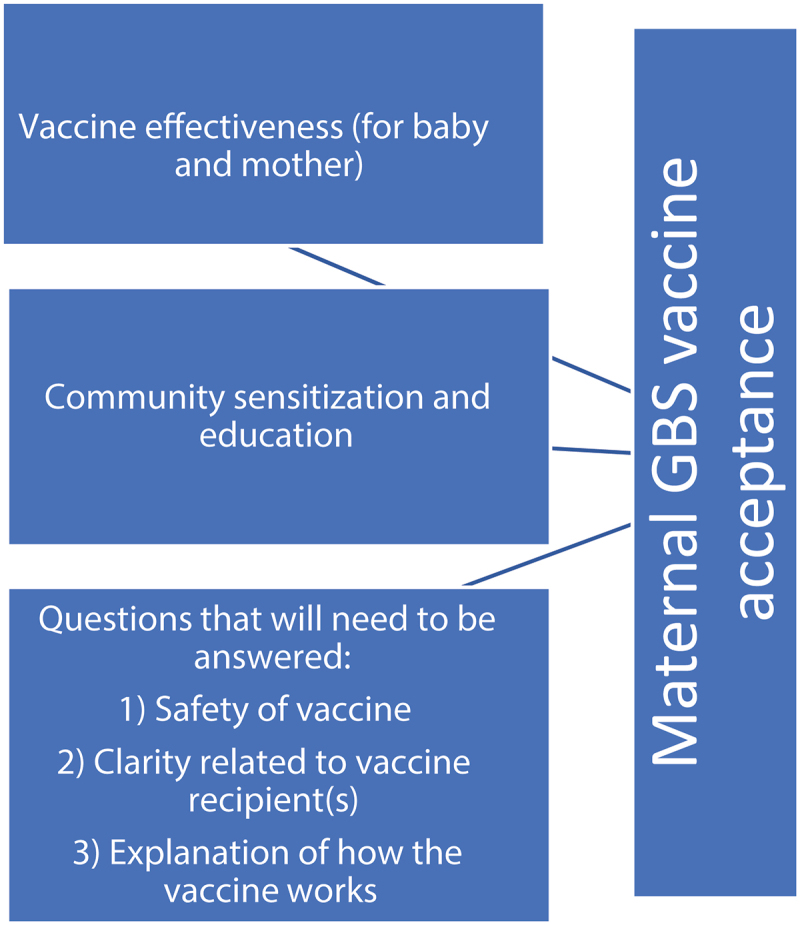
Antecedents and questions for maternal GBS vaccine acceptance.

Two key themes emerged from the data. The first focused on antecedents to maternal GBS vaccine acceptability. The most common antecedents focused on the vaccine’s ability to protect the baby and/or the mother, followed by community sensitization before the vaccine was available. The second key theme focused on questions that would need to be addressed before someone could accept a maternal GBS vaccine. Three key categories of questions were identified, including vaccine safety compared to vaccine benefits, who gets the vaccine, and how the vaccine works. See Figure 2 for a summary of key themes.

### Maternal GBS vaccine acceptability antecedents

We identified two key antecedents related to maternal GBS vaccine acceptability. The first focused on the vaccine’s ability to protect the baby and/or the mother, and the second focused on adequate community sensitization and education about the disease and the vaccine before a maternal GBS vaccine was available in their communities.

#### Protection of baby and/or mother

The most common reason why participants would accept a maternal GBS vaccine was protection against GBS for the baby and/or the mother. This lactating mother pointed out that she would get a maternal GBS vaccine given her desire to protect her baby and herself: “*I would absolutely take the vaccine because I want the best for my kid and myself*.” (Primipara, Molo Sub-County Hospital, Nakuru, rural). This lactating woman would accept the vaccine as she believed it would help her during birth and protect her baby: “*I will accept the vaccine, I am ready because it may help us to give birth well and also our babies not to be infected with diseases*.” (Primipara, Miritini Health Centre, Mombasa, urban). In a similar vein, this lactating woman was worried about not getting the vaccine and the effects this decision would have on her baby: “*I am thinking that maybe if I do not get the vaccination as I am supposed to, I can experience a lot of challenges, maybe like I give birth to a baby and then it passes on, also I may bleed a lot and lack energy in my body*.” (Primipara, Tudor Sub-County Hospital, Mombasa, urban). This pregnant woman conveyed that the benefits of a maternal GBS vaccine would be the most important factor for acceptance: “*There will not be with any issues for the mothers (with accepting a GBS vaccine). I have told you if it will benefit myself and my baby, if it will protect me and it will protect the fetus I am carrying, I will take it. Many times a woman who is pregnant should be the one to protect her baby so not many would refuse. If you want your baby to have a good life you will agree to go for the vaccine, other women will agree, everybody will accept because you will see it will help you*.” (Multigravida, primipara, Mwisho wa Lami Dispensary, Nakuru, rural). This pregnant woman echoed this sentiment related to the vaccine’s ability to protect the baby and the mother to inform decision-making: “*Once you have told us this vaccine is to prevent certain illnesses for the mother and baby, that we will understand, and accept and there will not be many questions. We will not ask many questions. Because you will have explained that I am getting this vaccine so that it can protect me and my baby from certain infections*.” (Multigravida, multipara, Jomvu Model Health Centre, Mombasa, urban).

#### Community sensitization

In addition to the protection a GBS vaccine can confer to the baby and the mother, the next most common factor for acceptance was community sensitization related to the disease and the vaccine to reduce negative effects related to the disease. This community member spoke about informing the community about the vaccine before the vaccine was available: “*As you have said your research is about a vaccine, it is very important that you know you cannot bring something in the market before informing the community on what it is*.” (Male partner of lactating mother, Nakuru Country Referral Hospital, Nakuru, rural). This community member asserted that community members would only have concerns if there was not adequate community education about the vaccine: “*I can have concerns because the government can announce a vaccine (has come to protect against) a certain disease, but let us say they did not do civic education, they did not send people or specialists to go around and give an idea that there is a vaccine or a certain disease, (all we have been told is) it will come later on*.” (Male partner of pregnant woman, Port Reitz Sub-County Hospital, Mombasa, urban). Finally, this pregnant woman asserted that education and community sensitization was important, coupled with social norms, or what peers did in related to accepting a maternal GBS vaccine: “*I think having to hear about it, it is something good to me, but then with time I need to trust it, and if it reaches majority of people and it works then I will be ok with it. If it comes tomorrow and I see people are getting it and they are ok and their children are ok, they are giving birth well, they have healthy babies, it has been there, I will go for it*.” (Primigravida, Mediheal Hospital, Nakuru, rural).

### Maternal GBS vaccine questions

We asked participants to imagine a scenario where a maternal GBS vaccine was approved and recommended by the Ministry of Health. Given this scenario, we asked participants what questions they had to accept the vaccine. Questions fell into three broad categories: vaccine safety compared to vaccine benefits, who gets the vaccine, and how the vaccine would work.

#### GBS vaccine questions: vaccine safety and vaccine benefits

Most questions focused on potential side effects from a maternal GBS vaccine. First, there were questions related to the effects of the vaccine on the fetus or the pregnancy, as this pregnant woman asked about how the vaccine could affect the baby: *“I would ask – when someone uses that vaccine, what will the child be like*?” (Primigravida, Rongai Health Centre, Nakuru, rural). This pregnant woman asked about how the vaccine could affect the baby or the mother in the context of a new vaccine: “*I would want to know if it has any negative effect on either the mother or the baby before I take it – I would want to know the side effects, of course. I would want to know how long it has been in use and the percentage of people that have been given the vaccine*.” (Primigravida, Mediheal Hospital, Nakuru, rural). This lactating mother asked about specific side effects she had experienced from other vaccines: “*I will have questions like does it have any side-effects in your body – can it affect me or the fetus I am carrying? Also, can it cause dizziness, heart racing?”* (Primipara, Tudor Sub-County Hospital, Mombasa, urban). This community member also commented on the need for communication to focus on side effects, and not just benefits: “*About that vaccine, I would want to know if it has been tested and what side effects does the vaccine have because most vaccines you find are helpful but they also have their various side effects and usually you find that those side effects are not mentioned. You always hear about the benefits but not the side effects*.” (Male partner of lactating woman, Tudor Sub-County Hospital, Mombasa, urban).

Second, there were questions related to availability of treatment if a side effect was experienced, as illustrated by this lactating woman: “*My question will be will it have side effects? The effects depend on ones’ body, you can be injected then it affects you and another person is injected but it does not affect them. Now if I am vaccinated with that vaccine and I get side effects, is there another medicine I can take to counter those side effects so that I can get well*?” (Multipara, Magongo Dispensary, Mombasa, urban). This pregnant woman had a similar question: “*My question is, that vaccine – can it give me some infection? What do I do if I get an infection*?” (Multigravida, multipara, Jomvu Model Health Centre, Mombasa, urban). This concern was also raised by this community member: “*Then when given the injection, if it affects me badly, can I come back here to the hospital for them to assist me*?” (Mother of pregnant woman, Kiptangwanyi Health Centre, Nakuru, rural). Finally, this question was also raised related to effects on the baby, as seen by this lactating woman: “*Yes, when I am vaccinated and the baby is affected because of the vaccine, when I go to a doctor, will they be able to treat my baby*?” (Multipara, Magongo Dispensary, Mombasa, urban).

#### GBS vaccine questions: who gets the vaccine

Participants had questions as to who would get the vaccine, such as this pregnant woman: “*Does the child also get it or the mother is also given? The mother? Just the child? Or also the mother*?” (Primigravida, Rongai Health Centre, Nakuru, rural). This lactating mother also wondered if the child would also get the vaccine, as well as dosing: “*How many times will she have to take this vaccination during a pregnancy? And when she delivers, will the child also get this vaccination*?” (Multipara, Mrima Health Centre, Mombasa, urban). This pregnant woman asked why her spouse would not receive the vaccine: *“I would ask the doctor – you have said that is a vaccine to protect me and my baby – what about my spouse? Why hasn’t my spouse been included, we are both involved in sex, and the pregnancy is ours. He may have an infection, or he may not. Or I start imagining that maybe he is the one who infected me. You see, now those are questions us women are likely to ask, and if they don’t ask the doctor, they ask themselves*.” (Multigravida, multipara, Jomvu Model Health Centre, Mombasa, urban).

#### GBS vaccine questions: how the vaccine works

Finally, there were questions about how the vaccine works to protect against GBS. Several participants questioned why a new vaccine would be introduced, suggesting that they were not aware of GBS, such as this lactating woman: “*Before we used to give birth well, our babies were fine. But now I have a question for the doctor – why has this new vaccine come and for what reasons? What does it treat? Why has it been added (to the other vaccines they tell us to take)? There must be a reason why you are adding this one because you have been vaccinating us with those others and they were treating us, so what has happened for you to add on this one*.” (Multipara, Magongo Dispensary, Mombasa, urban). This community member asked what the vaccine was treating, alluding to the fact that the participant was not aware of GBS: “*The first question I can ask about that vaccine is what does it treat or what disease does it prevent*?” (Male partner of pregnant woman, Port Reitz Sub-County Hospital, Mombasa, urban).

## Discussion

Development of maternal GBS vaccines for dual protection of the pregnant woman and her infant has been identified as a priority by the World Health Organization.^18^ However, GBS vaccines in and of themselves will not reduce morbidity and mortality, vaccination will. To our knowledge, little is known about attitudes toward maternal GBS vaccines; we could only find one study that empirically examined such attitudes. This study occurred in Australia and authors found that among pregnant women surveyed, the majority (95%) were either very likely or likely to receive a GBS vaccine.^19^ As such, our study is one of the first that can provide information for the purpose of informing demand generation efforts that will be crucial for vaccine acceptance. Authors of a 2013 commentary suggested that the speed of maternal GBS vaccine uptake will be dependent on public awareness.^20^ Results from our study suggest that pregnant and lactating women are open to the idea of a maternal GBS vaccine and articulated the importance of such a vaccine being able to protect the baby and the mother, as well as importance of community education and sensitization about the disease and vaccine before the vaccine was introduced. Participants also identified key questions that would need to be answered before accepting a vaccine, including vaccine safety compared to vaccine benefits, who gets the vaccine, and how the vaccine would work. These findings suggest that, to reduce vaccine hesitancy, efforts must be made to ensure that communities are adequately sensitized and provided adequate information about maternal GBS vaccines before the vaccine is available. In addition, to reduce vaccine hesitancy, it will be paramount to provide digestible and adequate answers to the vaccine-specific questions identified.

Participants also wanted to know whether a GBS vaccine would be given to a specific population, which may lead to questions about the effectiveness in pregnant populations with decreased immunity. Infections such as HIV and malaria lead to a decrease in IgG transfer to the infant and a previous GBS vaccine trial in Malawi and South Africa showed the vaccine was less immunogenic in HIV infected individuals.^21,22^ Therefore, it will be important for recipients with certain infection to understand these potential decreases in effectiveness.

A review that synthesized key elements for successful implementation of maternal immunization in low- and middle-income countries found that women’s vaccine decision-making is frequently motivated more by a desire to protect their babies than their own benefit.^23,24^ Others have noted the importance of women knowing the potential benefits of the vaccine for the mother and the child, as women who do not believe themselves or their infants to be at risk of disease are less likely to accept vaccination.^25,26^ This review also noted that education of pregnant women is key to vaccine acceptance, and understanding the multifactorial causes of vaccine hesitancy is essential to inform approaches targeted at the individual, provider, health system and national levels.^25^ Another review that sought to identify determinants of maternal immunization in developing countries found that health system factors such as financial and human resources were the biggest barriers, followed by provider-level barriers including poor attendance at antenatal clinics, and patient-barriers such as lack of knowledge related to maternal immunization.^26^ A study that explored provider perspectives on maternal immunization in Kenya suggested that improved patient and provider education, including material resources, and community engagement through religious and cultural leaders were essential for maternal vaccine acceptance.^27^ In another study conducted in Kenya that examined messaging for maternal immunization, authors found that including relevant and relatable factors in messaging were most likely to result in positive attitudes toward maternal vaccination.^28^ The results from our study add to this literature, specific to maternal GBS vaccine decision-making, and should be used to develop country-level readiness materials. Specifically related to vaccine hesitancy, ensuring that vaccine decision-makers and those that influence them have sufficient and relevant information about vaccine safety, are aware of their susceptibility to the disease, have an understanding related to the severity of the disease, and understand how the vaccine works and on what population will be critical for vaccine acceptance.

This study has limitations. This qualitative study was not designed to be generalizable. Social desirability bias is likely, as participants may have given us answers that they perceived we wanted to hear. As attitudes related to vaccines vary considerably by context and population, as we were only able to recruit participants from two counties, are findings are not representative. There are likely differences in attitude within communities. Findings were heavily dependent on the cross-sectional nature of the study. As the vaccine is not yet available, we asked participants about their feelings toward a hypothetical vaccine, which may be challenging for participants to conceptualize. Additionally, as GBS is not a well-known disease generally in Kenya, participants may not have been able to provide nuanced information about what information would be needed to influence maternal GBS vaccine acceptance, as they may have had limited knowledge about the disease itself. Nuance may have also been lost in language translation. This study also has strengths. Little is known about vaccine intentions among pregnant and lactating persons as well as community members related to future GBS maternal vaccines. Understanding the perspectives of this population will be crucial to create awareness and ultimately demand for new maternal vaccines, including the GBS vaccine, and affect subsequent acceptance. Additional research is needed to better understand other critical decision-making constructs among pregnant and lactating women, as these vary by location, context, and target population. Research should also take a holistic approach as pregnant and lactating women are influenced by many factors.

Realizing the potential individual- and community-level benefits of any maternal vaccine will require a multifactorial approach. This includes the strengthening of antenatal care services, including capacity related to maternal immunization delivery. This also includes ensuring that women and communities are aware of the harms of GBS as well as the safety and effectiveness of a maternal GBS vaccine.^11^ Our study contributes to informing this multifactorial approach by elucidating the attitudes and concerns of pregnant and lactating women as well as key influencers of these populations. Future research should seek to better understand health system factors that could affect maternal GBS vaccine delivery, as well as regulatory and policy factors that could affect vaccine access and equity.
